# Revisiting the Biological Behavior of *Salmonella enterica* in Hydric Resources: A Meta-Analysis Study Addressing the Critical Role of Environmental Water on Food Safety and Public Health

**DOI:** 10.3389/fmicb.2022.802625

**Published:** 2022-06-02

**Authors:** Alan Douglas de Lima Rocha, Rafaela Gomes Ferrari, Walter Esfrain Pereira, Laiorayne Araújo de Lima, Patrícia Emília Naves Givisiez, Andrea Isabel Moreno-Switt, Magaly Toro, Enrique Jesús Delgado-Suárez, Jianghong Meng, Celso José Bruno de Oliveira

**Affiliations:** ^1^Departamento de Zootecnia, Laboratório de Avaliação de Produtos de Origem Animal (LAPOA), Centro de Ciências Agrárias, Universidade Federal da Paraíba (UFPB), Areia, Brazil; ^2^Departamento de Ciências Fundamentais e Sociais, Centro de Ciências Agrárias, Universidade Federal da Paraíba (UFPB), Areia, Brazil; ^3^Escuela de Medicina Veterinaria, Facultad de Agronomía e Ingeniería Forestla, Facultad de Ciencias Biológicas, Facultad de Medicina, Pontificia Universidad Católica de Chile, Santiago, Chile; ^4^Laboratorio de Microbiologia y Probioticos, Instituto de Nutricion y Tecnologia de los Alimentos, Universidad de Chile, Santiago, Chile; ^5^Facultad de Medicina Veterinaria y Zootecnia, Universidad Nacional Autónoma de México, Ciudad de México, Mexico; ^6^Joint Institute for Food Safety and Applied Nutrition (JIFSAN), University of Maryland, College Park, College Park, MD, United States

**Keywords:** agriculture, epidemiology, foodborne pathogens, meta-analysis, one health, salmonellosis, systematic review

## Abstract

The increasing number of studies reporting the presence of *Salmonella* in environmental water sources suggests that it is beyond incidental findings originated from sparse fecal contamination events. However, there is no consensus on the occurrence of *Salmonella* as its relative serovar representation across non-recycled water sources. We conducted a meta-analysis of proportions by fitting a random-effects model using the restricted maximum-likelihood estimator to obtain the weighted average proportion and between-study variance associated with the occurrence of *Salmonella* in water sources. Moreover, meta-regression and non-parametric supervised machine learning method were performed to predict the effect of moderators on the frequency of *Salmonella* in non-recycled water sources. Three sequential steps (identification of information sources, screening and eligibility) were performed to obtain a preliminary selection from identified abstracts and article titles. Questions related to the frequency of *Salmonella* in aquatic environments, as well as putative differences in the relative frequencies of the reported *Salmonella* serovars and the role of potential variable moderators (sample source, country, and sample volume) were formulated according to the population, intervention, comparison, and outcome method (PICO). The results were reported according to the Preferred Reporting Items for Systematic Review and Meta-Analyzes statement (PRISMA). A total of 26 eligible papers reporting 148 different *Salmonella* serovars were retrieved. According to our model, the *Salmonella* frequency in non-recycled water sources was 0.19 [CI: 0.14; 0.25]. The source of water was identified as the most import variable affecting the frequency of *Salmonella*, estimated as 0.31 and 0.17% for surface and groundwater, respectively. There was a higher frequency of *Salmonella* in countries with lower human development index (HDI). Small volume samples of surface water resulted in lower detectable *Salmonella* frequencies both in high and low HDI regions. Relative frequencies of the 148 serovars were significantly affected only by HDI and volume. Considering that serovars representation can also be affected by water sample volume, efforts toward the standardization of water samplings for monitoring purposes should be considered. Further approaches such as metagenomics could provide more comprehensive insights about the microbial ecology of fresh water and its importance for the quality and safety of agricultural products.

## Introduction

Salmonellosis is a cosmopolitan disease caused by *Salmonella enterica*, a major pathogen causing human foodborne illness worldwide (Majowicz et al., 2010; Hendriksen et al., 2011; European Food Safety Authority [EFSA], 2016; Tack et al., 2019). *Salmonella* species are estimated to cause 93.8 million cases of gastroenteritis worldwide annually, leading to 59,100 deaths (Majowicz et al., 2010; Roth et al., 2018). In the United States (USA), gastroenteritis caused by non-typhoidal *Salmonella* only was estimated to affect approximately one million people annually resulting in approximately US$ 3.7 billion medical costs (Majowicz et al., 2010; Batz et al., 2012). *Salmonella* was associated with 33% of the foodborne illness cases registered in 2018 in ten sites covering 15% of the USA population and has been cited as the second most prevalent foodborne pathogen, preceded only by *Campylobacter* spp. (Tack et al., 2019).

The microbiological condition of water used in agriculture, regardless of the source, is crucial for the safety of agri-food products. Salmonellosis outbreaks have been associated with the use of contaminated water in agricultural settings (Harris et al., 2003; Walsh et al., 2014; Liu et al., 2018). As the gastrointestinal tract of vertebrates is generally considered the natural habitat of *Salmonella enterica*, the use of recycled water from animal production systems is usually considered a major risk factor for produce contamination (Abulreesh, 2012). However, *Salmonella* occurrence in water sources might go beyond short-term accidental findings determined by the transient presence of bacteria as a result of scattered fecal contamination events. Viability mechanisms can enable *Salmonella* organisms to successfully survive in natural aquatic environments for several months (Domingo et al., 2000; Liu et al., 2018). In laboratory, however, *Salmonella* has been observed to survive for up to 5 years in phosphate-buffered solution at room temperature (Liao and Shollenberger, 2003).

Although *Salmonella* can survive in a wide range of pH (4.05–9.5) and temperature (7–48°C) under controlled laboratory conditions (Cox et al., 2014), the natural environment associated with irrigation water sources such as rivers or lakes may impose challenging conditions for the long-term viability of *Salmonella.* Variations in physicochemical properties (temperature, salts, pH, oxygen), nutrient availability, interaction with other microorganisms, and exposure to UV radiation (Wilkes et al., 2011; Wanjugi and Harwood, 2013) have been shown to reduce *Salmonella* viability in water over time, generally up to 30 days (Steele and Odumeru, 2004). On the other hand, the production biofilm can facilitate the survival of *Salmonella* in water and aquatic invertebrates, such as free-living protozoa and vertebrate hosts (Sha et al., 2011; Liu et al., 2018; Chen et al., 2019). Furthermore, re-introduction of *Salmonella* into irrigation ponds should be also considered, as previously demonstrated for *Salmonella* Newport (Li B. et al., 2015). Re-introduction events are usually caused by animal waste contamination through sewage discharges, rainfall, or associated surface run-off events. Therefore, natural or non-recycled water sources such as rivers and irrigation canals have been shown to act as reservoirs of viable *Salmonella* (Baudart et al., 2000; Li et al., 2014; Martínez et al., 2017) and play a critical role as contamination sources of *Salmonella* and other microbes to fresh produce (Hanning et al., 2009), circulating back to humans and other animals (Li B. et al., 2015).

Despite the increasing number of studies reporting the presence of *Salmonella* in natural aquatic environments, there is no agreement on its average frequency and relative serovar representation across water sources. Because of this knowledge gap and the great importance of water for the sustainability of food production worldwide, this meta-analysis aimed at determining the weighted average proportion and between-study variance of *Salmonella* frequency in non-recycled water environments and the role of putative moderators affecting both the frequency and relative representation of serovars.

## Materials and Methods

Three sequential steps were performed by the authors in order to obtain a preliminary selection from identified abstracts and article titles: Identification of information sources, Screening and Eligibility. The selected articles were finally included in the study.

### Identification of Information Sources

The identification of putative information sources was guided by questions that were formulated according to the population, intervention, comparison, and outcome method (PICO) (Santos et al., 2007). The following questions were asked: What is the occurrence of *Salmonella* in aquatic environments? Are there differences in the presence of *Salmonella* between surface and groundwater? Which serovars are most prevalent in surface water? Which serovars are most prevalent in groundwater? Which serovars are present in both surface and groundwater? Are there differences in frequency and diversity of *Salmonella* serovars among countries? Could differences in the frequency and diversity of *Salmonella* be attributed to sample volume? Are there differences in presence and abundance related to seasonality?

A literature search was performed using Medical Subject Headings (MeSH) terms on Pubmed, Web of Science, and Embase databases. The search components are described below. The initial screening process was performed from April to November 2020. Further directed searches were carried out by checking the reference lists of relevant articles.

Search component 1 (SC1)—population: Water OR groundwater OR lake OR pond OR river.

Search component 2 (SC2)—intervention: *Salmonella* spp. OR *Salmonella enterica* OR Salmonella*.

After retrieving the search components results, the Boolean operator “AND” was used to combine SC1 and SC2.

### Screening

The research considered only papers in English published between 2015 and 2020 and duplicate articles were excluded. Editorials, letters, and Ph.D. thesis were also excluded. Based on the title and abstract contents, only articles presenting proper identification of the serovars isolated from surface or groundwater sources were selected.

### Eligibility

The eligibility assessment was performed after the complete analysis of the entire manuscript. For serotyping characterization, publications using the standard Kauffmann-Le Minor scheme were first selected, but some articles using serotyping through pulsed-field gel electrophoresis (PFGE) were also included. The exclusion of publications using rapid methods of *Salmonella* detection was justified by two key reasons. Firstly, publication reporting serovar identification provides more information for biological interpretation and therefore fit better the purpose of our study, as these publications can be used to respond all the focus questions. For instance, the assessment of the frequencies of *Salmonella* serovars could be biased by the inclusion of articles using primers targeting a small group of serovars. Secondly, although some rapid tests could provide higher sensitivity values for *Salmonella* detection compared to conventional microbiological culture (Cox et al., 2014), the comparative analysis could be biased by the large methodological variation represented by the numerous available tests, including commercial and in-house methods. Therefore, the publications considered in the present study described microbiological isolation methods performed according to standard methodologies such as BAM and AOAC, although minor differences existed, mainly in terms of types of media. Importantly, as the large number of serovars usually requires the use of a combination of culture media (Cox et al., 2014), there is possibility of bias in the comparative analysis of the serovars across the different studies. Finally, the results were reported according to the Preferred Reporting Items for Systematic Review and Meta-Analyses Statement (PRISMA) (Moher et al., 2015).

### Risk of Bias Assessment

Possible sources of bias included study inclusion/exclusion criteria and the impact of missing data, missing primary results, the chosen database, date, language, number of articles, and article type selected for this study.

### Statistical Analyses

Information regarding the identification of manuscripts (authors, publication year, country), total number of collected samples, number of positive samples, number of *Salmonella* serovars, sample source (superficial or ground water), and water sample volume were obtained and kept in excel spreadsheets. Frequencies of *Salmonella* serovars were calculated by dividing the number of positive samples of each serovar by the total number of collected samples. Spreadsheets containing the data used in this meta-analysis are available as Supplementary Material. Because of the existence of proportions outside the range of 0.2–0.8, the frequency values were logit-transformed before analysis. The summary effect size (i.e., the weighted average proportion) was obtained by fitting a random-effects model using the restricted maximum-likelihood estimator (RMLE), assuming there are within- and between-study variances across the studies. The estimates of summary proportions and their confidence intervals were visualized according to forest plot as proposed by Lewis and Clarke (2001). In order to assess the true variation in effect sizes (between-study variance), the study heterogeneity (τ^2^) was calculated and tested for significance according to *Q*-test at 95% probability. Heterogeneity was also quantified by *I*^2^ statistics as proposed by Higgins et al. (2003). When the effect sizes had high heterogeneity, we conducted a moderator analysis by means of meta-regression in order to investigate potential sources of systematic variation between the studies. Three potential moderators were investigated: (1) Sample source: surface water or groundwater; (2) Water sample volume: small (<999 mL) or large (≥1,000 mL); and (3) Sample origin: samples from countries with low (<0.8) or high (≥ 0.8) human development index (HDI), according to the latest Human Development Index Ranking (United Nations Development Programme [UNDP], 2020). We used China’s HDI for the reports from Taiwan. The analyses were performed using metafor package in R (Viechtbauer, 2010; RStudio Team, 2019; R Core Team, 2020). In addition, a decision tree was built by supervised machine learning using rpart package in R (Therneau et al., 2013), and the Pearson correlation coefficient between observed and estimated frequencies was calculated.

The influence of the moderators on the relative frequency of the reported *Salmonella* sorovars was verified with canonical correspondence analysis, using the vegan package in R (Oksanen et al., 2011). Diversity indexes Shannon and Pielou were also calculated for richness and evenness estimates, respectively.

The relative frequencies of serovars were submitted to cluster analysis of row and columns, based on binary distance and hierarchical clustering. Hierarchical clustering of the 26 articles condiering the relative frequencies of the serovars was built using average linkage was built from a binary distance matrix in R 4.11. The optimal number of clusters was defined according to the FOM (figure of merit) index using the clValid package in R (Brock et al., 2008). Heatmaps were obtained using the ComplexHeatmap package in R (Gu et al., 2016).

## Results

### Literature Search

A total of 1,723 articles were identified at PubMed, 1,277 at Web of Science, and 2,194 at Embase, totaling 5,194 papers. Of these, 1,972 duplicates or triplicates were detected and excluded. A total of 3,222 remaining publications were obtained after the exclusion of redundant papers. Most articles (*n* = 101) were excluded for not informing the proper identification of serovars.

**FIGURE 1:**
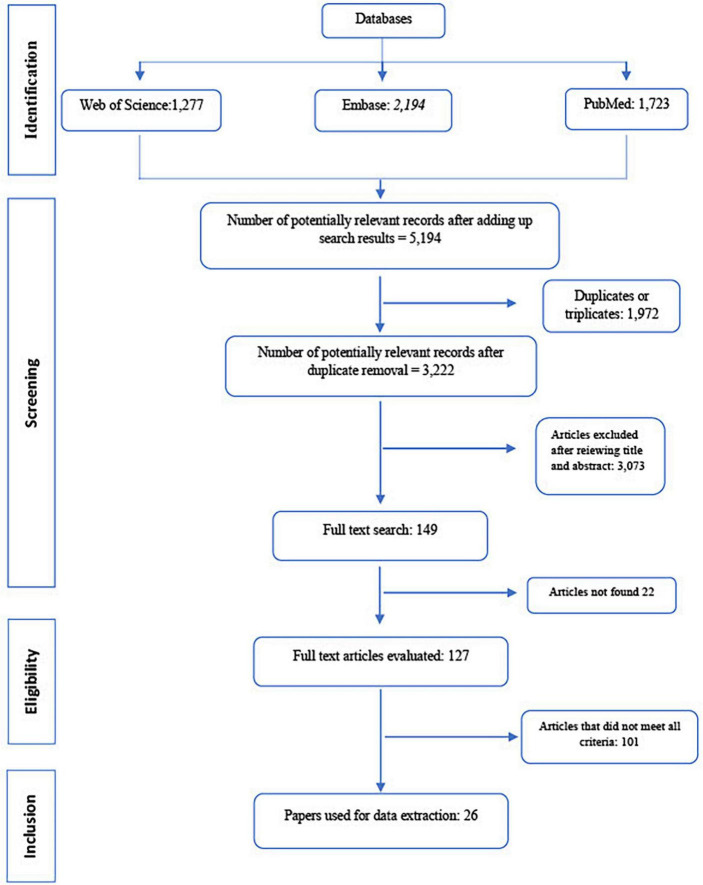
PRISMA flow diagram showing the sequential steps for articles selection and inclusion in the meta-analysis.

After titles and abstracts were read, 26 papers addressing both *Salmonella* and non-recycled environmental water were considered adequate and included in the present study (Figure 1). For statistical analysis, these 26 publications resulted in 29 observations as three papers reported *Salmonella* occurrence for both surface and groundwater.

A total of 148 different serovars were reported. Of these, 139 originated from surface water, from which 123 serovars were reported only in this water source. A total of 25 serovars were reported in groundwater, including nine serovars that were exclusively reported in this source. Only 16 serovars were reported in both sources.

The United States was identified as the country with the highest number of papers reporting the identification of *Salmonella* serovars (13), followed by Canada (3), and Taiwan (2). The remaining articles originated from Burkina Faso, China, Croatia, Ghana, Mexico, Spain, Sri Lanka, and Uganda.

Table 1 presents the relative frequency ranges of *Salmonella* serovars observed in non-recycled surface water and groundwater. Only serovars that were reported in at least five different studies were included in this table. Supplementary Tables 1, 2 provide detailed information about the occurrence of all reported *Salmonella enterica* serovars in the 26 publications.

**TABLE 1:** Relative representation of *Salmonella enterica* serovars associated with surface and groundwater sources that have been reported in peer-reviewed scientific publications addressing the occurrence of *Salmonella* in aquatic environments between the years 2015 and 2020 (Only serovars reported in at least five different studies are considered).

Serovar	Source	Relative representation, %	References
*S.* Newport	Surface water	1.14–58%	Bell et al., 2015; Hsu et al., 2015; Jokinen et al., 2015; Li B. et al., 2015; Maurer et al., 2015; Afema et al., 2016; Antaki et al., 2016; Bergholz et al., 2016; Topalcengiz et al., 2017; Harris et al., 2018; Ho et al., 2018; Song et al., 2018; Truitt et al., 2018; Callahan et al., 2019; Gu et al., 2019; Kadykalo et al., 2020; Mahagamage et al., 2020
	Groundwater	5.55 and 43.18%	Gu et al., 2019; Mahagamage et al., 2020
*S.* Typhimirium	Surface water	0.29–37.31%	Bell et al., 2015; Hsu et al., 2015; Jokinen et al., 2015; Maurer et al., 2015; Afema et al., 2016; Falardeau et al., 2017; Song et al., 2018; Truitt et al., 2018; Callahan et al., 2019; Gu et al., 2019; Díaz-Torres et al., 2020; Kadykalo et al., 2020; Mahagamage et al., 2020
	Groundwater	9.09–90.91%	Gu et al., 2019; Mahagamage et al., 2020; Stokdyk et al., 2020
*S.* Enteritidis	Surface water	0.75–50%	Jokinen et al., 2015; Li B. et al., 2015; Maurer et al., 2015; Afema et al., 2016; Bergholz et al., 2016; Falardeau et al., 2017; Santiago et al., 2018; Song et al., 2018; Callahan et al., 2019; Gu et al., 2019.
	Groundwater	100%	Kovačić et al., 2017.
*S.* Bareilly	Surface water	1.69–16.21%	Hsu et al., 2015; Li B. et al., 2015; Maurer et al., 2015; Harris et al., 2018; Ho et al., 2018; Truitt et al., 2018; Callahan et al., 2019; Gu et al., 2019; Mahagamage et al., 2020
	Groundwater	0%	
*S.* Thompson	Surface water	1.17–18.3%	Bell et al., 2015; Jokinen et al., 2015; Li B. et al., 2015; Maurer et al., 2015; Bergholz et al., 2016; Song et al., 2018; Truitt et al., 2018; Gu et al., 2019; Kadykalo et al., 2020
	Groundwater	36.36%	Gu et al., 2019
*S.* Infantis	Surface water	0.29–76%	Bell et al., 2015; Jokinen et al., 2015; Maurer et al., 2015; Bergholz et al., 2016; Truitt et al., 2018; Callahan et al., 2019; Kadykalo et al., 2020
	Groundwater	0%	
*S.* Saintpaul	Surface water	0.89–19.54%	Jokinen et al., 2015; Li B. et al., 2015; Maurer et al., 2015; Antaki et al., 2016; Topalcengiz et al., 2017; Harris et al., 2018; Truitt et al., 2018; Gu et al., 2019
	Groundwater	0%	
*S.* Agona	Surface water	1.83–86.67%	Hsu et al., 2015; Jokinen et al., 2015; Santiago et al., 2018; Song et al., 2018; Díaz-Torres et al., 2020; Kadykalo et al., 2020; Mahagamage et al., 2020
	Groundwater	0%	
*S.* Give	Surface water	1.15–12.31%	Jokinen et al., 2015; Maurer et al., 2015; Traoré et al., 2015; Bergholz et al., 2016; Harris et al., 2018; Callahan et al., 2019; Kadykalo et al., 2020
	Groundwater	3.85%	Dekker et al., 2015
*S.* Javiana	Surface water	0.44–24.89%	Bell et al., 2015; Jokinen et al., 2015; Li B. et al., 2015; Topalcengiz et al., 2017; Truitt et al., 2018; Gu et al., 2019; Mahagamage et al., 2020
	Groundwater	9.1%	Gu et al., 2019
*S.* Anatum	Surface water	0.85–9.52%	Maurer et al., 2015; Topalcengiz et al., 2017; Harris et al., 2018; Ho et al., 2018; Truitt et al., 2018; Callahan et al., 2019
	Groundwater	0%	
*S.* Hartford	Surface water	0.75–10.85%	Maurer et al., 2015; Antaki et al., 2016; Topalcengiz et al., 2017; Truitt et al., 2018; Callahan et al., 2019; Gu et al., 2019
	Groundwater	0%	
*S.* Kentucky	Surface water	0.19–21.21%	Jokinen et al., 2015; Maurer et al., 2015; Afema et al., 2016; Gu et al., 2019; Kadykalo et al., 2020; Mahagamage et al., 2020
	Groundwater	16.67%	Mahagamage et al., 2020
*S.* Muenchen	Surface water	0.89–14.84%	Jokinen et al., 2015; Li B. et al., 2015; Maurer et al., 2015; Antaki et al., 2016; Topalcengiz et al., 2017; Harris et al., 2018
	Groundwater	0%	
*S.* Rubislaw	Surface water	4.72–20.52%	Jokinen et al., 2015; Maurer et al., 2015; Antaki et al., 2016; Bergholz et al., 2016; Topalcengiz et al., 2017; Harris et al., 2018
	Groundwater	15.38%	Dekker et al., 2015
*S.* Senftenberg	Surface water	0.85–11.11%	Bell et al., 2015; Jokinen et al., 2015; Maurer et al., 2015; Traoré et al., 2015; Afema et al., 2016; Truitt et al., 2018
	Groundwater	0%	
*S.* Virchow	Surface water	1.49–8.1%	Li B. et al., 2015; Traoré et al., 2015; Afema et al., 2016; Ho et al., 2018; Santiago et al., 2018; Song et al., 2018
	Groundwater	0%	

**FIGURE 2:**
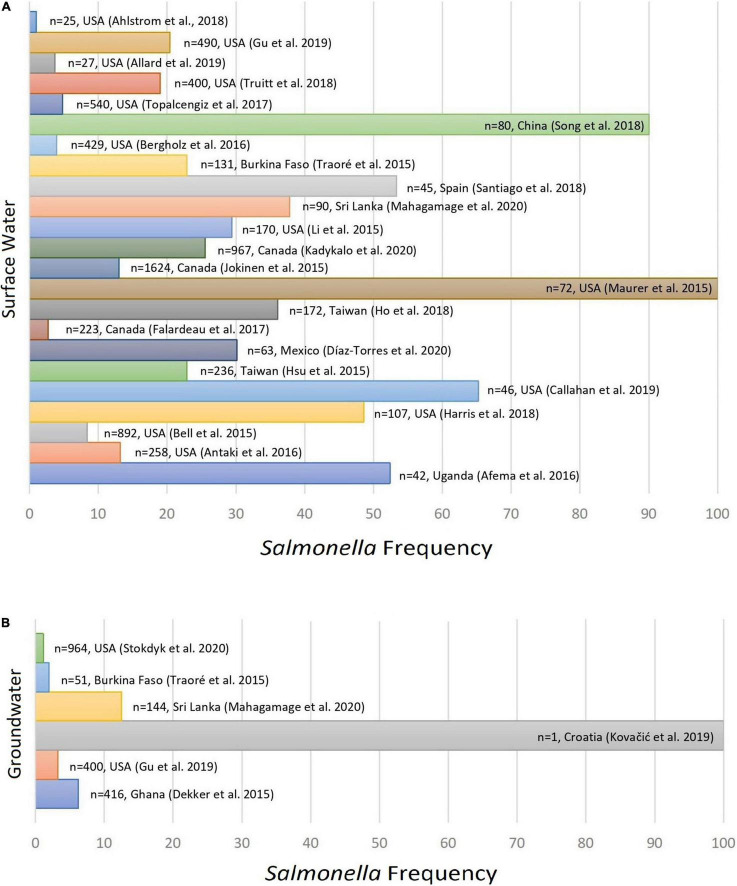
Frequency of *Salmonella enterica* serovars detected in non-recycled surface water **(A)** and groundwater samples **(B)** as per reported in 26 peer reviewed scientific publications between the years 2015 and 2020.

The frequency of *Salmonella enterica* in water samples varied from 1.14 (Stokdyk et al., 2020) to 100% (Maurer et al., 2015; Kovačić et al., 2017) as observed in Figure 2. The highest number of isolates reported in a single study (*n* = 247) was associated with surface water samples (Kadykalo et al., 2020), while the highest serovar diversity (35 different serovars) was observed by Jokinen et al. (2015).

Considering groundwater only (Table 1), the relative frequency of *Salmonella enterica* varied from 3.85 to 100%. The higher number of isolates for this type of water (*n* = 26) was reported by Dekker et al. (2015). Except for Kovačić et al. (2017), which reported the occurrence of *Salmonella* Enteritidis in a single sample (100%), the highest frequency of *Salmonella* and the greatest diversity of serovars were observed in a study conducted in Sri Lanka (Mahagamage et al., 2020). Importantly, although the study from Kovačić et al. (2017) refers to a single sample related to an outbreak investigation, no minimum sample size was predetermined as inclusion criteria for the present meta-analysis and therefore that study has been included in the present investigation.

**FIGURE 3:**
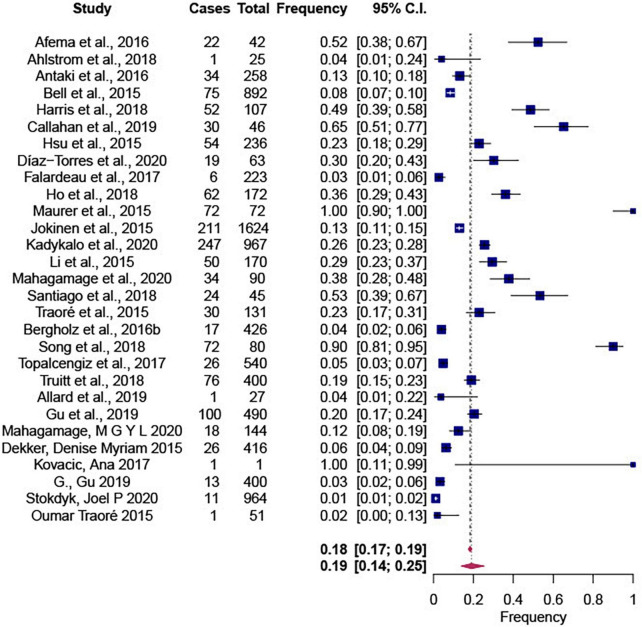
Forest plot showing the summary effect size of proportions of *Salmonella* frequencies in non-recycled water sources using 26 selected articles and 29 observations. This summary effect size was obtained in R 4.11 (package metafor) by fitting a random-effects model using the restricted maximum-likelihood estimator (RMLE). Heterogeneity parameters and statistics are indicated in the model.

The Forest plot showing the summary effect size of the *Salmonella* proportions in water is shown in Figure 3. According to our results, *Salmonella* frequency in non-recycled water sources was 0.19 [CI: 0.14; 0.25]. Although a significant (*P* < 0.0001) and high heterogeneity (τ^2^ = 0.0711; *I*^2^ = 99.72%) was observed, only source was identified as a significant mediator (*P* < 0.10) in the meta regression analysis. The descriptive average frequencies were 31.97 and 20.85% in surface water and groundwater samples, respectively, as shown in Supplementary Tables 1, 2.

**FIGURE 4:**
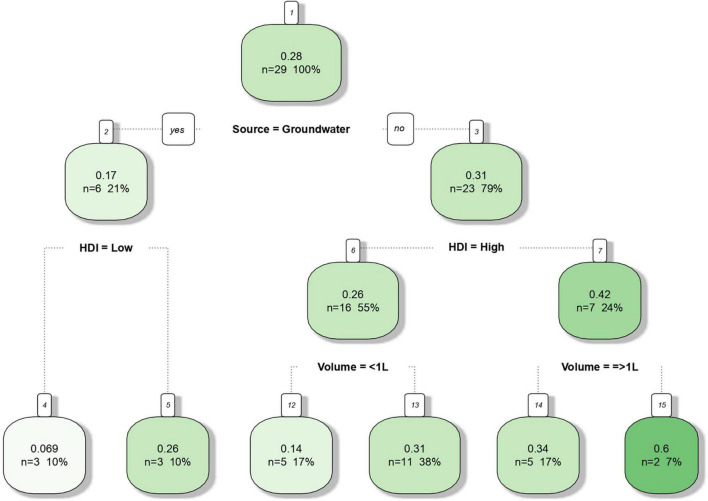
Decision tree predicting the frequency *of Salmonella* in non-recycled water sources in function of the moderator variables source (surface or groundwater), human development index (HDI) of the country from which the samples originated (high or low) and water sample volume (< 1 L or ≥ 1 L). The predictive algorithm has been built in R (package rpart) using meta-analysis data of 26 peer reviewed scientific publications between the years 2015 and 2020.

The decision tree (Figure 4) obtained by supervised machine learning resulted in a 0.48 Pearson correlation coefficient between observed and estimated frequencies. All three moderators (water source; HDI, and sample volume) were shown to affect *Salmonella* frequency in water but source was identified as the most relevant one. Estimate frequencies of 0.31 and 0.17% for surface and groundwater were obtained, respectively. Considering surface water only, samples from countries with lower HDI resulted in a higher *Salmonella* frequency (0.42) compared to developed regions (0.26). Sampling of small water volumes resulted in lower detectable *Salmonella* frequencies in both high and low HDI regions. The water sampling technique reported by the majority of the studies consisted of transporting determined volume of water to the laboratory for filtering. The use of less than 1 L water samples was reported by seventeen studies (65.38%), while other six studies (23.08%) described the use of 1–4 L water samples. Only two publications (7.69%) reported the use of *in situ* water filtration (10 L) by means of the modified Moore swab technique (MMS) (Allard et al., 2019; Callahan et al., 2019) and only a single study reported the use of *in situ* ultrafiltration (728 L) with commercial dialyzers (Stokdyk et al., 2020). Detailed information is shown in Supplementary Tables 1, 2.

According to the canonical correspondence analysis, both HDI and sample volume significantly (*P* < 0.0001) affected the relative frequencies of the 148 *Salmonella* serovars across the studies. The hierarchical clustering of the 26 publications considering the relative frequencies of the 148 *Salmonella* serovars is shown in Supplementary Figure 1. Five distinct clusters were observed. The larger cluster (2) is comprised only by studies from countries with high HDI and the majority of these studies reported the use of water samples with less than 1 L. On the other hand, all but one (16) study in the Cluster 1 originated from countries with low HDI. Two manuscripts (17 and 25) were not grouped in any cluster.

**FIGURE 5:**
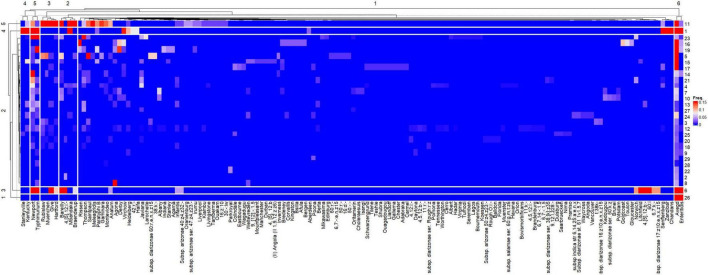
Heatmap of the relative frequencies of *Salmonella enterica* serovars isolated from surface and groundwater sources as per reported in 26 peer-reviewed scientific publications between the years 2015 and 2020. The heatmap was built in R (package ComplexHeatmap).

**FIGURE 6:**
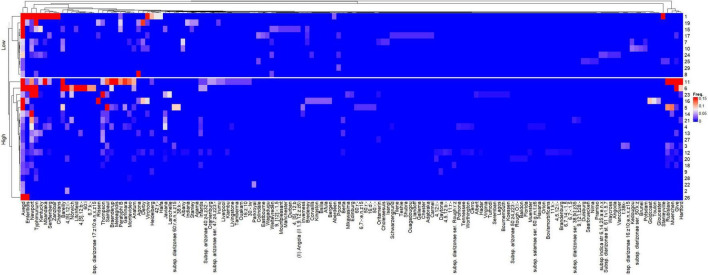
Heatmap of the relative frequencies of *Salmonella enterica* serovars isolated from non-recycled water sources according to the human development index (HDI) of countries associated with 26 peer-reviewed scientific publications between the years 2015 and 2020. The heatmap was built in R (package ComplexHeatmap).

**FIGURE 7:**
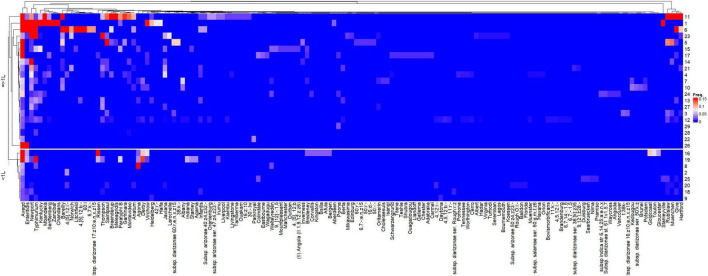
Heatmap of the relative frequencies of *Salmonella enterica* serovars isolated from non-recycled water sources according to the water sample volume used in 26 peer-reviewed scientific publications between the years 2015 and 2020. The heatmap was built in R (package ComplexHeatmap).

The heatmap representing the relative frequencies of *Salmonella* serovars according to the cluster analysis of rows (*y*) and columns (*x*) is shown in Figure 5. Cluster 1 (*x*) included the 135 less representative serovars, while cluster 5 (*x*) was comprised the most frequent serovars: *S.* Newport and *S.* Typhimurium, which were identified in 19 and 16 studies, respectively. Other frequent serovars were grouped in Clusters 2 (*S.* Barelly, *S.* Mbandaka, *S.* 4,[5],12:i:-, *S.* Braenderup), 3 (*S.* Rubislaw, *S.* Muenchen, *S.* Give, *S.* Hartford) and 4 (*S.* Kentucky, *S.* Stanleyville). Considering that the relative frequencies of the *Salmonella* serovars across studies are affected by HDI and volume, individualized heatmaps according to these moderators are shown in Figures 6, 7, respectively. The higher frequencies of some serovars such as *S.* Rubislaw, *S.* Muenchen, *S.* Give, *S.* Hartford, *S.* Rissen, *S.* Saintpaul, and *S.* Thompson across studies from high HDI countries is shown in Figure 6. According to Figure 7, some serovars were more frequently observed in studies using larger water samples, such as *S.* Newport, *S.* Typhimurium, *S.* Mbandaka, *S.* Braenderup, and *S.* Kentucky. On the other hand, *S.* Agona, *S.* Derby, and *S.* Virchow were more frequently observed in some studies using small volume water samples (8, 16, 19) than in studies using greater water volume samples.

There were no differences (*P* > 0.05) in the diversity of *Salmonella* serovars across water sources, HDI and volume as measured by Shannon and Pielou indexes for richness and evenness, respectively.

## Discussion

### Occurrence of *Salmonella* in Aquatic Environments

The overall weighted average proportion was 0.19 [CI: 0.14; 0.25] for positive water samples, suggesting that viable *Salmonella* organisms are frequently found in non-recycled water sources worldwide. The increasing number of human salmonellosis outbreaks associated with the consumption of fresh produce or industrialized plant-based products, including fruits, vegetables, legumes, grains, nuts, and seeds, has posed the question whether environmental water could play a role as potential *Salmonella* contamination source. The 127 peer-reviewed studies retrieved after the initial screening test of this systematic review confirms the increasing interest of the scientific community on this topic. These publications reported the occurrence of *Salmonella* from different aquatic environments under a wide range of conditions (Domingo et al., 2000; Bell et al., 2015; Li B. et al., 2015; Liu et al., 2018). Obviously, the capacity of *Salmonella* to survive and to adapt to challenging environmental conditions is a basic principle for the bacteria to fulfill their biological cycle (fecal-oral route of transmission), suggesting the existence of mechanisms to overcome stressors in aquatic and terrestrial microcosms. Long-term persistence of *S. enterica* in irrigation ponds has been previously reported (Greene et al., 2008; Luo et al., 2015), indicating that this pathogen is able to adapt to stressors in hydrological niches and highlighting the importance of assessing the quality of irrigation water regularly (Winfield and Groisman, 2003). However, little is known about the real ability of *Salmonella* to adapt and evolve in natural environments such as surface and groundwater sources.

### Are There Differences in the Presence of *Salmonella* Between Surface and Groundwater?

The amount of true heterogeneity observed in our study, represented by the between-study variance and expressed by the parameter (τ^2^), (Borenstein et al., 2005) was large and statistically significant, indicating the existence of systematic differences in effects across 26 articles used in the present meta-analysis. Interestingly, the *I*^2^ parameter varies from 0 to 100% and allows comparisons of the estimated heterogeneity across different meta-analysis studies. In the case of the present study, the observed *I*^2^ value was 99.72%. Therefore, there is evidence that the variance is determined by the existence of importance variable moderators. That said, we highlight the limited number of moderators (*n* = 3) investigated in the present meta-analysis that could act as sources of heterogeneity, as reflected by the lack of important information across all studies in the meta-analysis, for instance, the proximity of the sampled water of potential contamination sources (agriculture, livestock, sewage), psychochemical properties of water that might play an important role in the survival of *Salmonella* and competing organisms, the presence of other contaminants, characterization of wildlife in the proximity of the water bodies, among others.

The fact that the source of water was identified as a significant moderator for *Salmonella* frequency according to the meta regression analysis and was also identified as the most relevant moderator in the regression tree corroborates the hypothesis that viable *Salmonella* is more frequently recovered from surface water sources than groundwater. This finding was expected, considering the greater exposure of surface water sources to contaminants in general. Indeed, surface water is more easily exposed to discharge of sewage, inadequate agricultural, livestock and industrial run-offs, climatic events and visit of wild animals (Bergholz et al., 2016; Karkey et al., 2016; Toro et al., 2016; Gu et al., 2019; Jechalke et al., 2019). The latter is particularly noteworthy, since a wide range of *S. enterica* serovars have been extensively reported in wildlife (Maurer et al., 2015; Toro et al., 2016; de Souza et al., 2020).

The lower frequency of *Salmonella* in groundwater compared with surface water (0.17 vs.0.31, respectively) observed in our study corroborates previous findings (Abulreesh, 2012; Gu et al., 2019). Underground reservoirs have long been considered excellent sources of drinking water to human and animal populations, mainly because it is naturally filtered by the soil underlying rock formations. Although they usually provide superior microbial quality associated with lower microbial loads, the belief that groundwater is pure and no treatment is needed before consumption has been questioned (Li et al., 2018; Liu et al., 2018; Stokdyk et al., 2020) by the increasing number of studies reporting *Salmonella* contamination in groundwater (Dekker et al., 2015; Li X. et al., 2015; Palamuleni and Akoth, 2015). There are several possibilities of contamination of groundwater, even though some of them are sporadic. Quality can be compromised by insufficient well depth or during construction (Liu et al., 2018) and well pollution may result from events such as improperly functioning sewer systems, contaminated stormwater and agricultural run-off, especially after storms and floods (Gu et al., 2019). Contamination events can be sporadic or one-off, nevertheless, the water sources can become compromised for longer periods (Dekker et al., 2015). Some experimental studies showed that *Salmonella* can remain viable for periods longer than 100 days in water, and that viability is mainly affected by ambient temperature (Domingo et al., 2000; Ibrahim et al., 2019).

Importantly, there was a considerably higher number of studies on the occurrence of *Salmonella* in surface water (*n* = 23) compared with groundwater (*n* = 6). Possibly, the greater interest in assessing surface water is related to its relevance and economic importance for both rural and urban settings worldwide. In fact, the majority of reports in the present study originated from regions where surface water sources have been commonly used for irrigation purposes in agri-food production systems.

In summary, the occurrence of *Salmonella* in groundwater should not be neglected. Further studies addressing *Salmonella* contamination in groundwater are warranted as they could be particularly important in regions where irrigation practices depend on this type of water, such as semiarid settings.

### Which Serovars Are Most Prevalent in Surface Water and Ground Water?

Between 2015 and 2020, *S.* Newport was the most frequent serovar identified in both surface (464 isolates; 18.33%) and groundwater (20 isolates; 0.78%). Furthermore, Callahan et al. (2019) reported *S.* Newport as the most isolated serovar throughout the year. *S.* Newport infection rates have been stable over the decades, with approximately 750 confirmed cases per year in Europe (European Food Safety Authority [EFSA], 2016). Wild birds are considered important reservoirs as recurrent *S.* Newport outbreaks have been reported due to direct contamination of vegetables such as tomatoes, soil or irrigation water (Bell et al., 2015). The factors causing variations in *S.* Newport rates in the United States remain unknown (Crim et al., 2018).

*Salmonella* Typhimurium was the second most frequent serovar contaminating both surface water (9.56%) and groundwater (0.63%). This serovar has been one of the two leading serovars associated with human salmonellosis since 1990 (Herikstad et al., 2002). The persistence of this pathogen in freshwater microcosms has been associated with the expression of the *hil*A gene, a regulatory system for the expression of invasive *Salmonella* phenotypes, including the expression of the *ssp*C, *inv*F, and *org*A invasion genes (Nutt et al., 2003). Therefore, it is possible that strains circulating in environmental water sources could present increased virulence.

*S.* Thompson, also a frequent serovar, has been associated with sporadic salmonellosis outbreaks every year in different countries (Friesema et al., 2012; Gaulin et al., 2017; Suijkerbuijk et al., 2017; Eun et al., 2019). Under laboratory conditions, a 3 ppm chlorine water treatment induced the viable but not cultivable state in *S.* Thompson (Highmore et al., 2018), raising concerns about the efficacy of chlorine-based treatment of water for human consumption. Therefore, *S.* Thompson may be a potential pathogen of treated water for human consumption.

*S.* Javiana, *S.* Kentucky, and *S.* Rubislaw serovars have been also identified as frequent serovars contaminating non-recycled water sources. These serovars have been shown to play a role in human salmonellosis. The number of cases of *S.* Javiana has been dramatically increasing in the USA in the last decades (Centers for Disease Control and Prevention [CDC], 2013). It is worth noting that drinking water has been reported as an important source of human infection by *S.* Javiana (Clarkson et al., 2010; Mukherjee et al., 2019). *S.* Kentucky is involved in approximately 100 cases of human salmonellosis yearly in the United States (Centers for Disease Control and Prevention [CDC], 2016). Although it is not one of the leading serovars causing human salmonellosis, there is increasing concern with the emergence of multidrug resistance particularly associated with this serovar (Milton et al., 2018; Al-Gallas et al., 2021a,b). On the other hand, *S.* Rubislaw has been mainly detected in environmental samples (Maurer et al., 2015), and various free-living animals (Potter et al., 2011; Rush et al., 2020; Hernandez et al., 2021).

### Are There Differences in the Relative Frequency of *Salmonella* Serovars Among Regions?

According to our findings, there are indications that the origin of samples, as determined by the HDI index related to the country of origin, might contribute to both overall isolation frequency and relative distribution of *Salmonella* serovars. Based on the decision tree (Figure 4), the frequencies of *Salmonella*-positive samples in surface water were higher in countries with low HDI compared with countries with higher HDI (0.42 vs. 0.26, respectively). This finding could be explained by contamination events that are probably more frequent in developing regions as a result of improper sewage treatment and disposal. However, the opposite was observed for groundwater samples and frequency estimates were 0.069 and 0.26 for low and high HDI, respectively.

Further investigations should be conducted to address the differences in the relative frequencies of serovars between high and low HDI countries. Some serovars such as *S.* Muenchen, *S.* Give, *S.* Hartford, *S.* Rissen, *S.* Saintpaul, *S.* Rubislaw, and *S.* Thompson were highly frequent across studies from high HDI countries while others (*S.* Agona, *S.* Derby, *S.* Anatum) were more frequently observed in studies from low HDI countries. It is plausible to admit that the relative serovar frequencies across the regions depend on natural, social and economical drivers impacting the epidemiological and evolutionary aspects of *Salmonella enterica*, and therefore very difficult to be predicted.

Although meta-analysis indicated *Salmonella* Agona as a frequent serovar present in water samples from the low HDI countries included in our study, it is among the ten leading serovars associated with human salmonellosis in European countries, with 378–582 cases per year (Popa and Popa, 2021). Outbreaks of non-typhoidal salmonellosis associated with this serovar has been linked to fresh food consumption (Estrada-Acosta et al., 2014; Hassan et al., 2019; Ehuwa et al., 2021), such as papaya (Hassan et al., 2019) and tomato (Estrada-Acosta et al., 2014). Moreover, irrigation water is considered a major contamination source in agricultural settings (Estrada-Acosta et al., 2014). However, salmonellosis cases attributed to *S.* Agona have also been attributed to the consumption of contaminated processed foods such as peanut butter and infant formulae (Ehuwa et al., 2021).

### Can Differences in the Frequency and Diversity of *Salmonella* Be Attributed to Sample Volume?

Interestingly, water sample volume was shown to significantly affect the relative frequency of *Salmonella* serovars across the different studies. According to the decision tree (Figure 4), higher frequency of *Salmonella* was seen in larger water samples (≥ 1 L) from both high and low HDI countries. In high HDI countries, the frequencies were 0.31 vs. 0.14, while in low HDI countries, a greater difference was observed (0.34 vs. 0.6). Although water sample volume has been referred as critical factor for the recovery of *Salmonella enterica* from water, there are no previous reports directly assessing the role of water sample volume on *Salmonella* isolation frequency. This meta-analysis study suggests that water volume might play an important role on the recovery of viable *Salmonella* serovars in environmental water. Moreover, the relative frequency distribution findings reported in our study and visualized as a heatmap (Figure 7) indicate a higher recovery frequency of public health relevant *Salmonella* serovars when large water samples are used (≥ 1 L), such as *S.* Typhimuiurm, *S.* Newport, and *S.* Enteritidis.

Important aspects indicate that the occurrence of *S. enterica* in natural water sources is underestimated. Firstly, a considerable number of the studies in our investigation (27.58%) reported using small-volume samples (<1 L), which may compromise the microbiological recovery. Although there is a consensus toward the use of larger water samples to detect microorganisms present in low densities (Bisha et al., 2011; McEgan et al., 2013; Sbodio et al., 2013), there is a lack of studies comparing the real effect of water volume on the recovery of *Salmonella* serovars. Furthermore, conventional microbiological isolation is limited in terms of detection of viable but non-culturable bacteria (VBNC), i.e., organisms presenting a very low metabolic rate or state of dormancy (Lin et al., 2016). Problems in VBNC *Salmonella* cultivation and identification have been well documented (Oliver, 2005; Morishige et al., 2017). This condition might be of particular importance for *Salmonella* organisms in natural water environments, as bacteria may be subjected to many stressors.

Considering how the number of viable organisms might affect the accuracy of the conventional culture method, alternative techniques have been proposed to overcome cost and logistic problems associated with the transport of large volumes of water to laboratories. Among these, the modified Moore swab (MMS) stands out as a high efficient and low operating cost method alternative for *in situ* filtration of large sample volumes (usually 10 liters or more) (Sbodio et al., 2013; Sharma et al., 2020; Sikorski and Levine, 2020).

### Are There Differences in Presence and Abundance Related to Seasonality?

Due to the very limited number of publications with serovar identification covering long periods of time, no statistical analysis was performed to assess the relationship between *S. enterica* frequency and season or climatic condition. Seventeen of the twenty-six articles reported isolation of *S. enterica* from all or most of the samples collected during the entire experimental period. There is no substantial variation regarding the frequency of serovars throughout the different seasons of the year (Bell et al., 2015; Dekker et al., 2015; Jokinen et al., 2015; Maurer et al., 2015; Traoré et al., 2015; Afema et al., 2016; Bergholz et al., 2016; Falardeau et al., 2017; Topalcengiz et al., 2017; Harris et al., 2018; Ho et al., 2018; Santiago et al., 2018; Truitt et al., 2018; Callahan et al., 2019; Gu et al., 2019; Díaz-Torres et al., 2020; Stokdyk et al., 2020).

Four articles performed a single sampling per site (Hsu et al., 2015; Li B. et al., 2015; Kovačić et al., 2017; Allard et al., 2019). Despite having made multiple samplings from the same sites over time, one study still analyzed the data as a single set, because it focused on reporting the incidence of antibiotic resistance in the isolated strains and did not assess the variation of isolates over time (Kadykalo et al., 2020).

Two studies carried out in Colorado and Georgia (United States) showed higher isolation rates in different seasons, spring and autumn, respectively (Antaki et al., 2016; Ahlstrom et al., 2018). One study showed higher frequency in the rainy season, between spring and early summer (Song et al., 2018). Interestingly, Mahagamage et al. (2020) reported increased frequencies of *S. enterica* isolation from surface water in rainy seasons, while the contrary was observed for groundwater. Overall, the relationship between *Salmonella* isolation frequency and seasons of the year or dry or rainy period seems to depend on several local variables. Factors such as average temperature, predominant type of exploitation in the region (agriculture, livestock or industry), availability of water (scarcity or abundance, regardless of the season), type of source and location of the source (level of preservation or urbanization of the surroundings) seem to have a strong influence on water contamination levels throughout the year.

To better assess these relationships, it is necessary to include further studies on the effects of climatic factors over long periods of time.

In summary, this meta-analysis investigation established the expected frequency of *Salmonella* recovery from water samples. There is a higher recovery rate from surface water compared with ground water. The serovar representation across those samples can be affected by the investigated region and collected water sample volume, mainly for those serovars that are relevant in public health. Further conclusions about other putative important moderators were not possible because of the lack of information in the accessed studies. In this sense, we encourage longitudinal study designs and thorough serotyping that enable conclusions on seasonal variations or the effects of factors such as physicochemical parameters of water and special-temporal information. Furthermore, high throughput approaches such as metagenomics could provide invaluable information about complex relationships between *Salmonella* and other biotic factors. Given the importance of water quality for agri-food systems and the public health importance of *Salmonella*, it is extremely important to better understand this dynamics, so that more effective strategies to control and mitigate salmonellosis can be envisioned and designed.

## Data Availability Statement

The raw data supporting the conclusions of this article will be made available by the authors, without undue reservation.

## Author Contributions

AR: literature search, data analysis, and manuscript writing. RF: conceptualization, literature search, data analysis, and manuscript writing. WP: data analysis and manuscript revision. LL: data analysis and manuscript writing. PG, AM-S, MT, and ES: manuscript writing and revision. JM: conceptualization and manuscript revision. CO: conceptualization, manuscript writing, and manuscript revision. All authors contributed to the article and approved the submitted version.

## Conflict of Interest

The authors declare that the research was conducted in the absence of any commercial or financial relationships that could be construed as a potential conflict of interest.

## Publisher’s Note

All claims expressed in this article are solely those of the authors and do not necessarily represent those of their affiliated organizations, or those of the publisher, the editors and the reviewers. Any product that may be evaluated in this article, or claim that may be made by its manufacturer, is not guaranteed or endorsed by the publisher.

## Supplementary Material

Supplementary Figure 1 Hierarchical cluster analysis of 29 peer reviewed scientific publications between the years 2015 and 2020 considering the relative frequencies of reported *Salmonella* serovars. Hierarchical clustering using average linkage was built from a binary distance matrix in R 4.11.
