# Supplementary material for: Revisiting the Biological Behavior of Salmonella enterica in Hydric Resources: A Meta-Analysis Study Addressing the Critical Role of Environmental Water on Food Safety and Public Health
Source: Front Microbiol. 2022 Jun 2;13:802625. doi: 10.3389/fmicb.2022.802625 (PMC9201643; doi:10.3389/fmicb.2022.802625)
Supplement: Supplementary file 2 [file Table_1.DOCX]

**Table 1.** Frequency of serovars of *Salmonella enterica* subsp. *enterica* associated with surface water sources that have been reported in peer reviewed scientific publications between the years 2015 and 2020.

| Serovars | Country | Prevalence (%) | Volume of water analyzed | References |
| --- | --- | --- | --- | --- |
| 42:r:-; Aberdeen; Zanzibar; Virchow; Typhimurium; Stanleyville; Senftenberg; Chandans; Enteritidis; Paratyphi B; Newport; Haifa; Heidelberg; Mbandaka; Kentucky | Uganda | 22/42 (52.38%) | 1 L | (Afema et al., 2016) |
| 4,[5],12:i:- | USA | 1/25  (4%) | 50 mL | (Ahlstrom et al., 2018) |
| t:38k-; subsp. diarizonae 60:r,e,n,x,z15; subsp. diarizonae 16:z10:e,n,x,z15; Saintpaul; Rubislaw; Newport; Hartford; Muenchen | USA | 34/258 (13.18%) | 1 L | (Antaki et al., 2016) |
| 4,5,12:i:-; Worthington; Typhimurium; Thompson; Tennessee; Braenderup; Senftenberg; Cerro; Schwarzengrund; Poona; Gaminara; Newport; Muenster; Havana; Mbandaka; Infantis; Livingstone; Javiana | USA | 75/892 (8.41%) | 1 – 4 L | (Bell et al., 2015) |
| 16:d:-; 38:k:-; 50:-:-; 50:r:-; 6,7:-:e,n,z15; 60:r:-; Anatum; Bareilly; Braenderup; subsp. diarizonae 60:r,e,n,x,z15; Saintpaul; Rubislaw; Gaminara; Give; Newport; Muenchen; Meleagridis; Inverness | USA | 52/107 (48.6%) | 2 L | (Harris et al., 2018) |
| 4,[5],12:b:-; 4,[5],12:i:-; 6,7:k:-; 60:I; Anatum; Typhimurium; Bareilly; Braenderup; subsp. diarizonae 17:z10:e,n,x,z15; Subsp. arizonae 40:z4,z32:-; Enteritidis; Give; Norwich; Newport; Hartford; Infantis; Litchfield | USA | 30/46 (65.22%) | 10 L | (Callahan et al., 2019) |
| Agona; Weltevreden; Albany; Typhimurium; Bareilly; Bredeney; Choleraesuis; Derby; Potsdam; Oranienburg; Newport; Montevideo; Mbandaka; Isangi; Kedougou | Taiwan | 54/236 (22.88%) | 2 L | (Hsu et al., 2015) |
| Agona; Weltevreden; Typhimurium | Mexico | 19/63 (30.16%) | 25 mL | (Díaz-Torres et al., 2020) |
| Typhimurium; Daytona; Enteritidis; Heidelberg | Canada | 6/223 (2.69%) | 532 mL | (Falardeau et al., 2017) |
| Albany; Anatum; Virchow; Bareilly; subsp. diarizonae ser. 50:k:z; Brunei; Derby; Potsdam; Newport; Kedougou | Taiwan | 62/172 (36.05%) | 2 L | (Ho et al., 2018) |
| 16:z:10; 30:-:lw; Anatum; Typhimurium; Bareilly; Thompson; Tamberma; Braenderup; subsp. arizonae ser. 47:z4,z23:-; Subsp. arizonae 40:z4,z32:-; Senftenberg; Saintpaul; Rubislaw; Enteritidis; Paratyphi B; Gaminara; Ouakam; Give; Oranienburg; Newport; Hartford; Muenchen; Heidelberg; Montevideo; Meleagridis; Mbandaka; Infantis; Livingstone; Liverpool; Inverness; Kiambu; Kentucky | USA | 72/72 (100%) | 1 L | (Maurer et al., 2015) |
| 11:k:-; 11:r:-; 4,12:i:-; 4,5,12:b:-; 4,5,12:i:-; 6,7,14:-:1,5; 6,7:-:1,5; Agona; Albany; Indiana; Braenderup; Worthington; Typhimurium; Thompson; Berta; Tennessee; Bovismorbificans; subsp. diarizonae 60:r,e,n,x,z15; Brandenburg; Cerro; Senftenberg; Saintpaul; Daytona; Derby; Rubislaw; Enteritidis; Oranienburg; Give; Hadar; Newport; Muenster; Muenchen; Heidelberg; Infantis; Kiambu; Kentucky; Javiana | Canada | 211/1624 (12.99%) | 1 L | (Jokinen et al., 2015) |
| 4,5,12:b:-; Agona; Typhimurium; Thompson; Berta; Give; Newport; Heidelberg; Infantis; Kentucky | Canada | 247/967 (25.54%) | 1 L | (Kadykalo et al., 2020) |
| Bareilly; Enteritidis; Javiana; Montevideo; Muenchen; Saintpaul; Thompson; Virchow; Inverness; Newport | USA | 50/170 (29.41%) | 10 L | (Li et al., 2015) |
| 4,[5],12:b; 9,[12]:-:1,5; 1,9,12:z:z6; Agona; Weltevreden; Typhimurium; Bareilly; Bredeney; Stanley; Durban; Paratyphi B; Newport; Mountpleasant; Manchester; Litchfield; Kentucky; Javiana | Sri Lanka | 34/90 (37.78%) | 2,5 L | (Mahagamage et al., 2020) |
| Afula; Agona; Virchow; Toulon; Bergen; Bredeney; Bsilla; Corvallis; Derby; Rissen; Enteritidis; Gloucester; Goldcoast; Kingston | Spain | 24/45 (53.33%) | 200 mL | (Santiago et al., 2018) |
| Adabraka; Carmel; Schwarzengrund; Wagadugu; Virchow; Tilene; Teshie; Bredeney; Shubra; Senftenberg; Chester; Colindale; Eastbourne; Rissen; Poona; Galiema; Ouagadougou; Give; Llandoff | Burkina Faso | 30/131 (22.9%) | 1 L | (Traoré et al., 2015) |
| Enteritidis; Thompson; subsp. diarizonae ser. Rough:r:z; Rubislaw; Give; Newport; Infantis | USA | 17/429 (3.96%) | 250 mL | (Bergholz et al., 2016) |
| Agama; Agona; Virchow; Typhimurium; Thompson; Stanley; Derby; Rissen; Enteritidis; Paratyphi B; Newport; Indiana; Irumu | China | 72/80 (90%) | 500 mL | (Song et al., 2018) |
| 4,12:i:-; 4,5,12:i:-; Anatum; Baildon; Subsp. arizonae 50:z4,z23:-; Saintpaul; Rubislaw; Rough O:i:-; Florida; Hartford; Newport; Muenchen; Javiana | USA | 26/540 (4.81%) | 500 mL | (Topalcengiz et al., 2017) |
| 9,12:I,z28:-; Anatum; Typhimurium; Bareilly; Thompson; Berta; subsp. diarizonae ser. 38:[k]:z35; Senftenberg; Saintpaul; Norwich; Newport; Hartford; Infantis; Javiana | USA | 76/400 (19%) | 1 L | (Truitt et al., 2018) |
| Parkroyal | USA | 1/27 (3.7%) | 10 L | (Allard et al., 2019) |
| Albert; Edinburg; Enteritidis; Hadar; Tumodi; Virginia; Typhimurium; Bareilly; Thompson; Bournemouth; Seremban; Saintpaul; Oritamerin; Norwich; Newport; Hartford; Mikawasima; Larochelle; Lagos; Koessen; Kentucky; Javiana | USA | 100/490 (20,41%) | 4 L | (Gu et al., 2019) |
