# Supplementary material for: Revisiting the Biological Behavior of Salmonella enterica in Hydric Resources: A Meta-Analysis Study Addressing the Critical Role of Environmental Water on Food Safety and Public Health
Source: Front Microbiol. 2022 Jun 2;13:802625. doi: 10.3389/fmicb.2022.802625 (PMC9201643; doi:10.3389/fmicb.2022.802625)
Supplement: Supplementary file 3 [file Table_2.DOCX]

| Serovar | Country | Prevalence (%) | Volume of water analyzed | References |
| --- | --- | --- | --- | --- |
| Colindale; Duisburg; Give; Nima; Pramiso; Rubislaw; Saarbruecken; Stanleyville | Ghana | 26/416 (6,25%) | 200 mL | (Dekker et al., 2015) |
| Javiana; Newport; Oritamenrin; Thompson; Typhimurium | USA | 13/400 (3,25%) | 4 L | (Gu et al., 2019) |
| Enteritidis | Croatia | 1/1 (100%) | 1 L | (Kovačić et al., 2017) |
| Subsp. diarizonae st. 61 1v1,5,7; Angoda; Corvallis; Kentucky; Mbandaka; Newport; Poona; subsp indica str 6,14,25:a:e,n,x; Typhimurium; Vancouver; Waycross; Weltevreden | Sri Lanka | 18/144 (12,5%) | 2,5 L | (Mahagamage et al., 2020) |
| Poona | Burkina Faso | 1/51 (1,96%) | 1 L | (Traoré et al., 2015) |
| Montevideo; Typhimurium | USA | 11/964 (1,14%) | 728 L | (Stokdyk et al., 2020) |

**Table 2.** Frequency of serovars of *Salmonella enterica* subsp. *enterica* associated with groundwater sources that have been reported in peer reviewed scientific publications between the years 2015 and 2020.
